# Emotional and instrumental feeding practices of Dutch mothers regarding foods eaten between main meals

**DOI:** 10.1186/1471-2458-14-171

**Published:** 2014-02-17

**Authors:** Lieke GM Raaijmakers, Dorus WM Gevers, Dorit Teuscher, Stef PJ Kremers, Patricia van Assema

**Affiliations:** 1 Department of Health Promotion, NUTRIM School for Nutrition, Toxicology and Metabolism, Maastricht University Medical Centre +, Maastricht University, PO Box 616, 6200 MD Maastricht, The Netherlands; 2 Department of Human Biology, NUTRIM School for Nutrition, Toxicology and Metabolism, Maastricht University Medical Centre +, Maastricht University, PO Box 616, 6200 MD Maastricht, The Netherlands

**Keywords:** Parenting practices, Sweets and snacks, Primary school children

## Abstract

**Background:**

To assess how much of a public health problem emotional and instrumental feeding practices are, we explored the use of these feeding practices in a sample of Dutch mothers regarding their child’s food intake between main meals.

**Methods:**

A cross-sectional questionnaire study was conducted among 359 mothers of primary school children aged 4–12 years. The questionnaires were completed online at home.

**Results:**

Of the mothers, 29.5% reported using foods to reward, 18.1% to punish and 18.9% to comfort their child. Mothers most frequently offered energy-dense and nutrient-poor products such as candy in the context of emotional and instrumental feeding practices. The use of these practices was associated with a lower age of both mother and child and a higher educational level of the mother. Mothers living in neighborhoods with intermediate socioeconomic position used the practices less often than mothers from low and high socioeconomic position neighborhoods.

**Conclusions:**

Our results show that mothers in our sample mainly used unhealthy products in the context of instrumental and emotional feeding practices. Research into the association between these practices and children’s dietary intake is warranted, since the use of unhealthy products in the context of these practices may not necessarily lead to an increased consumption of these products. Findings regarding the frequency of use of these practices among specific subgroups can be used to carefully determine the target population for interventions and tailor the content of interventions to specific target group characteristics. Besides examining associations between personal and family characteristics and the use of emotional and instrumental feeding practices, attempts should be made to understand parents’ reasons for using them.

## Background

The high consumption of unhealthy foods such as sweets, snacks and soft drinks between main meals among young people is a major lifestyle problem in Western society and has important implications for public health [1-3]. These between-meal snacks are often high in calories, sugar or fat and have a low nutritional value, resulting in unhealthy dietary intakes [4]. This can contribute to dental problems [4], the rapidly growing problem of overweight and obesity, and the risk of developing chronic diseases at a later age [1,4,5].

The Netherlands Nutrition Centre (NNC) recommends a maximum of four snacking breaks between the three main meals, with the exception of water and coffee and tea without milk or sugar [6]. The NNC also recommends eating core products such as fruit or vegetables between main meals and not exceeding certain age- and gender-specific amounts of kcals from non-core products like snacks and soft drinks [7]. The average energy intake from foods and drinks consumed between main meals among Dutch children aged 4–13 years is two to three times higher than recommended [2,3]. National Food Consumption Survey data show that 24 to 40% (the percentage varies between age groups) of children aged 7–14 years had 8 or more food consumption occasions a day [8].

An important category of factors influencing children’s dietary intake is that of parent-related factors, including general parenting styles [9,10] and food-specific parenting practices [11]. The present study focused on the food-specific parenting practices of using food in between meals to regulate a child’s emotions (emotional feeding) and using food as a reward or withholding food as a punishment (instrumental feeding).

There are widely held beliefs that emotional and instrumental feeding practices are undesirable parental behaviors [12]. The foods that are typically offered in the context of these practices are thought to be palatable and energy-dense [13,14]. Previous studies found that using food as a reward for consuming other foods may increase the preference for the reward food and decrease healthy preferences in the long run [15,16]. One possible explanation may relate to the overjustification hypothesis, which says that children develop a decreased preference for the rewarded behavior (e.g. eating fruit) [17]. Furthermore, Wardle et al. argue that emotional and instrumental feeding can both encourage the child to associate eating with cues other than hunger, thereby increasing the risk of eating at times when there is no physiological need [12], possibly through a reduced ability of the child to perceive signs of hunger and satiety [18]. This inability of children to identify whether they are hungry or suffering from other discomfort and the tendency to overeat in response to emotional arousal is defined as emotion eating [19]. The evidence that emotional and instrumental feeding practices are indeed undesirable has been inconsistent. On the one hand, a study exploring memories about food showed that obese patients reported emotional feeding practices of their parents during their youth [20]. In a similar qualitative study, a few adults who scored high on binge eating and restraint eating recalled instrumental feeding in childhood [21]. Furthermore, previous research into the relationship between emotional and instrumental feeding and children’s food intake has found instrumental feeding to be positively associated with children’s snacking behavior [22] and more specifically a higher sweets consumption [23]. In addition, Kröller and Warschburger found that rewarding significantly decreased the intake of fruit and vegetables among children aged 4–6 years [24]. By contrast, however, no associations between instrumental feeding and fruit, vegetable and soft drink consumption [23] or with problematic foods in general (including fastfood, snacks, sweets and soft drinks were found) [24]. In spite of the relatively weak evidence, experts have suggested that parents should for the time being be advised not to use emotional and instrumental feeding [12].

To assess how much of a public health problem emotional and instrumental feeding practices are, it is important to know the effects of these practices on foods actually eaten between main meals, but it might be even more important to first examine the actual use of these potential parental risk behaviors and the type of foods that are offered in this context within specific populations. Therefore, this study aimed to explore the use of emotional and instrumental feeding practices among a sample of mothers of primary school children aged 4–12 years in the Netherlands, and to find out what types of products are offered in this context. Assessing the association between maternal use of instrumental and emotional feeding practices and children’s actual intake was beyond the scope of our study.

So far, only one study assessing the use of instrumental and emotional feeding has been conducted in a Dutch population [21] and it found mean scores of 1.5 and 1.3 on a 5-point Likert scale for instrumental and emotional feeding practices among parents of children aged 6–7 years. A number of studies outside the Netherlands did assess the use of emotional and instrumental feeding, but there might be cultural differences in the prevalence of this type of parental behavior [25]. Moreover, these studies measured the prevalence by asking parents to indicate on 5- or 6-point scales to what extent they used emotional and instrumental feeding practices [12,13,16,23-25]. Only mean scores were reported; the studies by Saxton et al. [13] and Wardle et al. [12] reported scores of 1.9 and 2.0 for emotional feeding, and 2.3 for instrumental feeding, using five-point scales. Moreover, reported mean scores were not always accompanied by information about the scoring system (e.g. from 1 to 5 or from 0 to 4) [16,25]. These mean scores do not provide much information on the proportion of parents that use these practices. Therefore, we decided to use dichotomous measures to assess the use of emotional and instrumental feeding.

To date, mixed results have been reported regarding subgroup differences in the use of emotional and instrumental feeding practices. Emotional feeding was found to be more prevalent among higher income parents [26], mothers of overweight children [27], lower educated mothers [13], mothers with a higher level of emotional eating [12,25] and parents in less deprived neighborhoods [28]. Instrumental feeding was found to be more prevalent among mothers with a higher level of external eating [12,25] and among mothers with lower levels of family income [29]. Other studies found no associations between emotional and instrumental feeding practices and the BMI of the mother [12] or that of the child [12,16], though a recent study in Australia found that overweight and obese girls were more likely to be rewarded with sweets for good behavior [30].

This study explored the use of emotional and instrumental feeding practices among parents of primary school children aged 4–12 years in the Netherlands, and aimed to find out which type of products are offered in this context. The research questions were: (1) How many mothers use emotional and/or instrumental feeding practices? (2) For which foods do mothers use emotional/instrumental feeding practices? (3) Are there differences in emotional and instrumental feeding practices between different subgroups (in terms of demographics, perceived body size and familiarity with the national recommendations)?

## Methods

### Participants and recruitment

A cross-sectional design was used in which 680 mothers of primary school children aged 4–12 years were recruited for participation. Only mothers were recruited for the present study, since they usually have the primary responsibility for feeding the children [31]. Data collection took place through a research agency, which invited members of their existing research panel for online questionnaire surveys to fill in the web survey. The members received points for participation in surveys, which they could exchange for gift vouchers. The agency was chosen because of the comprehensiveness of their research panel and the presence of individuals from a range of socio-economic positions (SEP) included in the panel. The panel was stratified based on region (postal code) and educational level of the mother. A group of mothers of primary school children was selected from this panel, with a distribution in terms of region and educational level that was comparable to that in the Dutch population, based on information from Statistics Netherlands. Ethical approval for this study was not required under Dutch law [32].

### Instrument development

The items used to assess instrumental and emotional feeding were created by the authors for the purpose of this study. The use of instrumental and emotional feeding practices was assessed using general dichotomous items to cover the full construct, unlike previous studies, which measured the practices on 5- or 6-point scales (e.g. [12,13,23,25]).

In depth-interviews with four mothers of primary school children were conducted to inform the questionnaire development. The printed questionnaire was piloted by four mothers, and the questionnaire was also discussed with three health promotion research experts, after which after adjustments were made. Subsequently, the digital questionnaire was piloted by two mothers and six health promotion research experts.

### Measures

Mothers were asked to answer all child-related questions for their youngest child attending primary school. *Personal and family characteristics* included family situation (living together with the biological father of all children, living together with a partner who is not the biological father of all children, living alone with children, other); country of birth of mother and father; weight, height and perceived body size of the mother (I am too thin, I am a little bit too thin, I have a normal weight, I am a little bit too heavy, I am too heavy); level of education of the mother; and number of children attending primary school. Mothers were also asked to report their child’s age, gender, perceived body size and whether their child attended childcare. The research agency provided data on the mother’s age, postal code and total number of children.

*Familiarity with the national recommendation regarding snacking breaks between main meals* was measured with two items. One item assessed whether respondents knew the recommendation regarding the number of snacking breaks children are allowed to have (viz. a maximum of four snacking breaks between the three main meals, with the exception of water and coffee and tea without milk or sugar). If they answered yes, the mothers were asked to write down the recommended number.

*Emotional feeding practices* were measured with a set of items that started with one dichotomous question asking mothers whether they used foods to comfort their child (no/yes). If mothers indicated doing so, they were shown a list of 26 products and asked to specify which products they used for this practice (yes/no) (potato chips, nuts and/or savory snacks; cookies; pastry and cake; ginger cake; bread; crackers and biscuit rusks; breadsticks; chocolate; candy bars; candy; French fries; deep fried snacks; cheese; sausage; ice cream; yoghurt and soft curd cheese; pudding; fruit; raw vegetables; water; soft drinks; fruit juice; milk; chocolate milk and yoghurt drink; other drinks; other).

*Instrumental feeding practices* were measured with two dichotomous questions asking mothers (1) whether they withheld certain products from their children between main meals to punish them and (2) whether they used products in between main meals to reward their child. If the respondents reported instrumental feeding practices, they were presented with the same list of 26 products, and were asked to indicate which products they used for this practice (yes/no).

### Data analysis

Body Mass Index (BMI) of the mother was calculated using weight and height, and divided into categories in accordance with the standard BMI classification by the World Health Organization [33]. The child’s ethnicity was used as a dichotomous variable (Dutch origin or not) based on the definition of ethnic minorities used by Statistics Netherlands, i.e., having at least one parent born abroad [34]. Postal code was recoded as 'socioeconomic position (SEP)’, based on a factor score (range -4 [high] to 4 [low]) calculated from four SEP indicators for all Dutch postal code areas, i.e. mean income, percentage of low income households, percentage of residents without a paid job and percentage of households with average or low education [35]. This factor score has a mean of 0.0 and scores of 1 and -1 should be interpreted as 1* the standard deviation, etc. [35]. The SEP variable was divided into tertiles (low-intermediate-high). Educational level was recoded as a categorical variable (low level [primary or basic vocational education], intermediate level [secondary vocational school or high school] and high level [higher professional education or university]), according to the definitions used by Statistics Netherlands [36]. Mother’s age was categorized into five groups: (1) ≤ 30, (2) 30–35, (3) 35–40, (4) 40–45, (5) ≥ 45. Child’s age was categorized into three groups: (1) ≤4–5, (2). 6–9; (3). 10–12. The cut-off points for these classifications were data-driven, and we aimed at obtaining groups with similar sizes.

Data were analyzed using SPSS 20.0. A multiple logistic regression analysis using the Enter method was conducted to determine associations between response (i.e. whether or not mothers participated) and the mother’s age, postal code, and number of children living at home as independent variables, in order to assess selective response. Multiple logistic regression analyses using the Enter method were performed to assess associations between using emotional or instrumental feeding (punishment and reward) practices and the mother’s age, educational level, SEP, BMI, perceived body size and familiarity with the recommendations, use of child care, and the child’s age, gender, perceived body size, and ethnicity as independent variables. Contrasts of the correlates of the above-mentioned dependent variables were tested by repeating the logistic regression analyses using a different reference group for each categorical independent variable each time. P-values <0.05 were considered to indicate statistical significance.

## Results

### Response and participants

Of the 680 mothers in the random sample, 24 were excluded since they did not have children in primary school. Of the remaining mothers, 359 (56%) responded within the requested one-week period. No significant differences in demographic characteristics were found between participants and non-participants.

Table 1 shows the personal and family characteristics of the participants and their youngest child. The mean age of the mothers was 38.4 (SD 5.6) years. Compared to the Dutch population, low-educated mothers were somewhat underrepresented, while highly educated mothers were somewhat overrepresented. Mean BMI was 25.8 kg/m^2^ and nearly half of the mothers (47.9%) would be classified as overweight or obese, which is representative of the general Dutch population [37]. The mean age of the children whom the mothers reported on was 7.0 (SD 2.7) and close to 90% of the children were of Dutch ethnicity, meaning that ethnic minorities were slightly underrepresented.

**Table 1:** Demographic, socioeconomic and other characteristics of mothers and children included in the study (N = 359)

**Characteristics**	**Mean (SD)**	**%**
***Characteristics of the mother***		
**Age**	38.4 (5.6)	
**≤** 30		7.8
30–35		22.0
35–40		33.1
40–45		27.6
≥ 45		9.5
**Educational level**^**1**^		
Low		21.4
Intermediate		46.8
High		31.8
**SEP**^**2**^		
Low		33.4
Intermediate		31.8
High		34.8
**BMI (kg/m**^**2**^**) in categories**^**3**^	25.8 (5.1)	
Underweight ≤ 18,49 kg/m^2^		3.1
Normal 18,5–24,99 kg/m^2^		48.9
Overweight 25–29,99 kg/m^2^		28.2
Obese 30 kg/m^2^ and above		19.8
**Perceived body size**		
(A little bit) too thin		4.8
Normal weight		33.1
(A little bit) too heavy		62.1
**Family situation**		
Living together with biological father of all children		76.9
Living together with partner (not biological father of all children)		8.1
Living alone with children		12.8
Other		2.2
**Number of children living at home**		
1		22.6
2		56.3
3		16.4
4 or more		4.7
**Child care use**		
0 days		61.6
≥ 1 day		38.4
**Familiar with NNC recommendation**		
Yes		20.3
No		79.7
***Characteristics of the child***		
**Age in years**	7.0 (2.7)	
≤ 4		21.7
5		17.8
6		11.4
7		11.1
8		7.8
9		6.7
10		7.5
11		8.4
≥12		7.5
**Gender**		
Boys		55.2
Girls		44.8
**Perceived body size**		
(A little bit) too thin		20.9
Normal weight		73.3
(A little bit) too heavy		5.8
**Ethnicity**^**4**^		
Dutch origin		89.7
Non-Dutch origin		10.3

^1^Educational level (low [primary or basic vocational education], intermediate [secondary vocational school or high school] and high [higher professional education or university]) [36].

^2^Socioeconomic position (SEP) is based on a factor score (range -4 [high] to 4 [low]) calculated from four indicators of SEP for all Dutch postal code areas, i.e. mean income, percentage of low-income households, percentage of residents without a paid job and percentage of households with intermediate or low education [35]. The SEP was divided into tertiles with cut-off points: (low [2.62–0.27], intermediate [0.27– -0.44], high [-0.44– -2.68]).

^3^According to standard BMI classification by the World health Organization [33].

^4^Non-Dutch origin: having at least one parent born abroad [34].

One out of five mothers reported being familiar with the recommendations for the number of snacking breaks a child is allowed to have between main meals. Of these 73 mothers, 17.8% (N = 13) answered in line with the NNC recommendations (i.e. four moments), meaning that 3.6% of all mothers were familiar with the recommendations (not tabulated).

### Emotional and instrumental feeding

Table 2 shows that the use of foods to reward a child was reported more often (29.5%) than the use of foods to comfort (18.1%) or punish the child (18.9%). With regard to subgroup differences in the use of emotional and instrumental feeding, we found that older mothers and intermediate-SEP mothers were less likely to use foods in between main meals to reward or comfort their child than younger mothers and high- and low-SEP mothers (Table 2). Mothers with an intermediate educational level were less likely to use foods to reward than highly educated mothers. Mothers were less likely to use foods to punish or reward older children than younger children, and less likely to use foods to reward girls than boys. No significant differences regarding the use of emotional and instrumental feeding were found for mother’s perceived body size, use of child care, child’s perceived body size, child’s ethnicity, and mother’s familiarity with the recommendations.

**Table 2:** Percentages of mothers using emotional and instrumental feeding practices in the total group and demographic subgroups (N = 359)

	**Instrumental feeding**		**Emotional feeding**
**Groups**	**Punishment (%, CI)**	**Reward (%, CI)**	**Comfort (%, CI)**
**Total group**	18.9 (15–23)	29.5 (25–34)	18.1 (14–22)
**Age of mother**			
(1). ≤ 30 (n = 28)	28.6 (11–46)	39.3 (20–59)	32.1 (14–51)
(2). 30–35 (n = 79)	26.6 (17–37)	44.3 (33–56)	26.6 (17–37)
(3). 35–40 (n = 119)	15.1 (9–22)	23.5 (16–31)	17.6 (11–25)
(4). 40–45 (n = 99)	14.1 (7–21)	21.2 (13–29)	8.1 (3–14)
(5). ≥ 45 (n = 34)	18.2 (6–35)	30.3 (16–49)	18.2 (4–31)
**Sign. contrasts**		3,4 < 2	4 < 1,2,3
**Education of mother**^**1**^			
(1). Low (n = 77)	19.5 (10–29)	26.0 (16–36)	19.5 (10–29)
(2). Moderate (n = 168)	16.8 (11–23)	24.0 (18–31)	16.2 (10–22)
(3). High (n = 114)	21.1 (13–29)	39.5 (30–49)	20.2 (13–28)
**Sign. contrasts**		2 < 3	
**SEP**^**2**^			
(1). Low (n = 120)	21.7 (14–29)	33.3 (25–42)	22.5 (15–30)
(2). Intermediate (n = 114)	20.2 (13–28)	21.9 (14–30)	12.3 (6–18)
(3). High (n = 125)	15.2 (9–22)	32.8 (24–41)	19.2 (12–26)
**Sign. contrasts**		2 < 1,3	2 < 1,3
**BMI of mother (kg/m**^**2**^**)**^**3**^			
(1). Underweight (n = 11)	36.4 (2–70)	27.3 (4–59)	0.0
(2). Normal weight (n = 175)	17.1 (12–23)	32.6 (26–40)	17.7 (12–23)
(3). Overweight (n = 101)	20.0 (13–29)	25.0 (17–34)	22.0 (14–30)
(4). Obese (n = 71)	18.3 (9–28)	26.8 (16–37)	16.9 (8–26)
**Sign. contrasts**			
**Age of child**			
(1). ≤4–5 (n = 142)	24.6 (17–32)	39.4 (31–48)	20.4 (14–27))
(2). 6–9 (n = 133)	20.3 (13–27)	27.1 (19–35)	19.5 (13–26
(3). 10–12 (n = 84)	7.1 (2–13)	16.7 (9–25)	11.9 (5–19)
**Sign. contrasts**	3 < 1,2	3 < 1	
**Gender of child**			
(1). Boys (n = 198)	18.7 (13–24)	33.3 (27–40)	20.7 (15–26)
(2). Girls (n = 161)	19.3 (13–25)	24.8 (18–32)	14.9 (9–20)
**Sign. Contrasts**		2 < 1	

Note: this table only shows the independent variables for which significant contrasts were found; significant contrasts were retrieved by comparisons made among the numbered subgroups by repeating the logistic regression analysis using a different reference group for each independent variable each time.

^1^Educational level (low [primary or basic vocational education], intermediate [secondary vocational school or high school] and high [higher professional education or university]) [36].

^2^Socioeconomic position (SEP) is based on a factor score (range -4 [high] to 4 [low]) calculated from four indicators of SEP for all Dutch postal code areas, i.e. mean income, percentage of low income households, percentage of residents without a paid job and percentage of households with intermediate or low education [35].

^3^According to standard BMI classification by the World health Organization [33].

The type of food most frequently used to withhold as a punishment, to reward or comfort children was candy (Table 3). In addition, withholding cookies and chocolate was frequently used to punish while ice cream was often used to reward or comfort the child. On average, 5 food products were used to punish the child, while foods used to reward and to comfort the child included 3 and 2 products, respectively.

**Table 3:** Most frequent applications of emotional and instrumental feeding practices for relevant product categories

**Practices**	**Most frequently applied using**				
**Instrumental feeding**					
Products withheld as punishment	Candy (82.4%)	Cookies (52.9%),	Candy bars (41.2%)	Ice cream (36.8%)	Pastry and cake (26.5%)
		Chocolate (52.9%)	Chips, nuts and savory snacks (41.2%)		
Products used as reward	Candy (59.4%)	Ice cream (43.4%)	Chocolate (26.4%)	Chips, nuts and savory snacks (25.5%)	Cookies (24.5%)
**Emotional feeding**					
Products used to comfort	Candy (66.2%)	Ice cream (30.8%)	Cookies (29.2%)	Chocolate (15.4%)	Chips, nuts and savory snacks (12.3%)

## Discussion

This study explored the use of emotional and instrumental feeding practices among parents of primary school children aged 4–12 years in the Netherlands, and aimed to find out which type of products are offered in this context. A substantial proportion of the mothers used emotional and instrumental feeding practices. Mothers most frequently offered energy-dense and nutrient-poor products such as candy, cookies, chocolate, ice cream and candy bars in the context of their emotional and instrumental feeding practices, while core products like bread, fruit and vegetables were least often used in this context. Comparison of our results with those of previous studies is limited by the fact that these studies reported the use of the practices in mean scores, which are difficult to compare with our dichotomous measures. Moreover, previous studies did not assess the practices specifically in relation to consumption in between meals. Our findings indicate that mothers mainly used unhealthy products in the context of emotional and instrumental feeding practices. This underlines the need to further investigate the potential negative effects of these practices on children’s food intake and health, and to develop interventions.

We found emotional and instrumental feeding practices to be associated with mother’s age, SEP, and educational level, and the child’s age and gender. In contrast to earlier studies, which reported that highly educated mothers make less use of food as a reward for positive behavior and make less use of emotional feeding [13], our results showed that mothers with an intermediate educational level were less likely to use foods as reward than highly educated mothers. Furthermore, older mothers appeared to be less likely to use foods to reward or comfort their child. We did not find an association between emotional and instrumental feeding practices and the BMI of the mother or child, which is in line with previous studies [12,25]. These studies were, however, conducted among parents with young children (3–7 years) and it might be that the parent’s behavior had not yet impacted on their children’s weight [16].

The majority of the mothers were not familiar with the NNC recommendation about the maximum number of snacking breaks children are advised to have between main meals. Moreover, the majority of mothers who reported being familiar with the recommendations were wrong about the recommended maximum number of snacking breaks. Since we did not find relevant associations between familiarity with the recommendation and the use of instrumental or emotional feeding practices, our study does not indicate that it is important for parents to know these recommendations.

A strength of the current study is that we used a sample that was representative of the Dutch population in terms of BMI and regional variation, which increases the potential for generalizing the results of the current study to the entire population of Dutch mothers of primary school children. Some limitations must, however, be acknowledged. The validity and reliability of the composite instrument we used have not yet been established. However, the use of our dichotomous measures to assess the use of instrumental and emotional feeding practices seems to provide some added value compared to previous studies that reported mean scores, since it forced participants to decide whether or not they used the feeding practices, and as such clearly revealed how many parents were actually using the practices. A limitation of these dichotomous measures is that we were not able to quantify the use of these feeding practices, which could be relevant especially in the case of negative consequences of these practices. Another limitation is that our results are based on self-reported data, which may have been subject to bias (e.g. through social desirability). In addition, we asked mothers to report only on their youngest child, while almost 80% of the mothers reported having more than one child, so this may have biased our results. The sample included relatively larger numbers of younger children, resulting in a relatively low mean age. The age limitation probably means that the use of instrumental feeding was overestimated, as fewer mothers reported using foods to punish or reward older children.

## Conclusion

Our findings indicate that research into the harmfulness of emotional and instrumental feeding practices is warranted. Our results provide a first indication of specific subgroups in which these practices are more likely to be used (viz. younger mothers, intermediate SEP mothers and higher educated mothers). These findings can be used to inform the development of future interventions to reduce these potentially harmful parental behaviors among the identified risk groups. Besides examining associations between personal and family characteristics and the use of emotional and instrumental feeding practices, we also consider it useful to understand parent’s reasons for using them. For instance, Campbell, Crawford, and Hesketh explored Australian parents’ views on their children’s food choices and gained some insight into parent’s thoughts about using foods as rewards [38]. It is possible that parents perceive rewarding with food as an effective strategy to have their child eat more healthy foods, since rewarding may be effective in the short term [39]. Therefore interventions to reduce these undesirable behaviors should also address long-term consequences.

## Competing interests

The authors declare that they have no competing interests.

## Authors’ contributions

LR drafted the manuscript. All authors made substantial contributions to the conception and design of the study. LR, DT and PvA made substantial contributions to data acquisition. All authors made substantial contributions to the analysis and interpretation of the data and have read and approved the final version of the manuscript.

## Pre-publication history

The pre-publication history for this paper can be accessed here:

http://www.biomedcentral.com/1471-2458/14/171/prepub

## References

[B1] Currie C Roberts C Morgan A Smith R Settertobulte W Samdal O Barnekow Rasmussen V Young people’s health in context. Health behaviour in school-aged children (HBSC) study: international report from the 2001/2002 survey 2004 Geneva: WHO

[B2] TNO Voeding Basisrapportage en tabellen voedselconsumptiepeiling 1997-1998 en gegevens op het niveau van voedingsmiddelen en voedingsstoffen over een periode van tien jaar. (Basic report and tables on the 1997-1998 food consumption survey and results on the level of food products and nutrients over a 10-year period) http://www.zuivelengezondheid.nl/tno/Start.htm

[B3] Ocké MC Van Rossum CTM Fransen HP Buurma EJM de Boer EJ Brants HAM Niekerk EM Van der Laan JD Drijvers JJMM Ghameslou Z Dutch National Food Consumption Survey - Young Children 2005/2006 2007 Bilthoven: National Institute for Public Health and the Environment

[B4] World Health Organization Diet, Nutrition and the Prevention of Chronic Diseases 2003 Geneva: WHO 12768890

[B5] Ministry of Health Welfare and Sport Preventienota Kiezen voor gezond leven (Prevention memorandum: Choosing a healthy lifestyle) 2006 The Hague: Ministry of Health Welfare and Sport

[B6] Netherlands Nutrition Centre Richtlijnen voedselkeuze (Guidelines on food choice) 2009 The Hague: Netherlands Nutrition Centre

[B7] Netherlands Nutrition Centre Tabel hoeveelheden calorieën voor extra’s (Table of amounts of calories for extras) 2009 The Hague: Netherlands Nutrition Centre

[B8] van Rossum CTM Fransen HP Verkaik-Kloosterman J Buurma-Rethans EJM Ocke MC Dutch National Food Consumption Survey 2007–2010: Diet of children and adults aged 7 to 69 years 2011 Bilthoven: National Institute for Public Health and the Environment

[B9] Kremers SP Brug J de Vries H Engels RC Parenting style and adolescent fruit consumption Appetite 2003 41 1 43 50 10.1016/S0195-6663(03)00038-2 12880620

[B10] Brown R Ogden J Children’s eating attitudes and behaviour: a study of the modelling and control theories of parental influence Health Educ Res 2004 19 3 261 271 10.1093/her/cyg040 15140846

[B11] Birch L Savage JS Ventura A Influences on the development of children’s eating behaviours: from infancy to adolescence Can J Diet Pract Res 2007 68 1 1 56 PMC2678872 19430591

[B12] Wardle J Sanderson S Guthrie CA Rapoport L Plomin R Parental feeding style and the inter-generational transmission of obesity risk Obes Res 2002 10 453 462 10.1038/oby.2002.63 12055321

[B13] Saxton J Carnell S van Jaarsveld CH Wardle J Maternal education is associated with feeding style J Am Diet Assoc 2009 109 5 894 898 10.1016/j.jada.2009.02.010 19394477

[B14] Evans A Seth JG Smith S Harris KK Loyo J Spaulding C Van Eck M Gottlieb N Parental feeding practices and concerns related to child underweight, picky eating, and using food to calm differ according to ethnicity/race, acculturation, and income Matern Child Health J 2011 15 7 899 909 10.1007/s10995-009-0526-6 19771501

[B15] Newman J Taylor A Effect of a means-end contingency on young children’s food preferences J Exp Child Psychol 1992 53 2 200 216 10.1016/0022-0965(92)90049-C 1578198

[B16] Carnell S Wardle J Associations between multiple measures of parental feeding and children’s adiposity in United Kingdom preschoolers Obesity (Silver Spring) 2007 15 1 137 144 10.1038/oby.2007.513 17228041

[B17] Birch LL Birch D Marlin DW Kramer L Effects of instrumental consumption on children’s food preference Appetite 1982 3 2 125 134 10.1016/S0195-6663(82)80005-6 7137991

[B18] Faith MS Scanlon KS Birch LL Francis LA Sherry B Parent–child feeding strategies and their relationships to child eating and weight status Obes Res 2004 12 11 1711 1722 10.1038/oby.2004.212 15601964

[B19] Kroller K Jahnke D Warschburger P Are maternal weight, eating and feeding practices associated with emotional eating in childhood? Appetite 2013 65 25 30 23380038 10.1016/j.appet.2012.11.032

[B20] Brink PJ Ferguson K Sharma A Childhood memories about food: the Successful Dieters Project J Child Adolesc Psychiatr Nurs 1999 12 1 17 25 10.1111/j.1744-6171.1999.tb00037.x 10347427

[B21] Puhl RM Schwartz MB If you are good you can have a cookie: how memories of childhood food rules link to adult eating behaviors Eat Behav 2003 4 3 283 293 10.1016/S1471-0153(03)00024-2 15000971

[B22] Sleddens EF Kremers SP De Vries NK Thijs C Relationship between parental feeding styles and eating behaviours of Dutch children aged 6–7 Appetite 2010 54 1 30 36 10.1016/j.appet.2009.09.002 19747513

[B23] Vereecken CA Keukelier E Maes L Influence of mother’s educational level on food parenting practices and food habits of young children Appetite 2004 43 1 93 103 10.1016/j.appet.2004.04.002 15262022

[B24] Kröller K Warschburger P Associations between maternal feeding style and food intake of children with a higher risk for overweight Appetite 2008 51 1 166 172 10.1016/j.appet.2008.01.012 18342396

[B25] de Lauzon-Guillain B Musher-Eizenman D Leporc E Holub S Charles MA Parental feeding practices in the United States and in France: relationships with child’s characteristics and parent’s eating behavior J Am Diet Assoc 2009 109 6 1064 1069 10.1016/j.jada.2009.03.008 19465189

[B26] Musher-Eizenman D Holub S Comprehensive Feeding Practices Questionnaire: validation of a new measure of parental feeding practices J Pediatr Psychol 2007 32 8 960 972 10.1093/jpepsy/jsm037 17535817

[B27] Baughcum AE Powers SW Johnson SB Chamberlin LA Deeks CM Jain A Whitaker RC Maternal feeding practices and beliefs and their relationships to overweight in early childhood J Dev Behav Pediatr 2001 22 6 391 10.1097/00004703-200112000-00007 11773804

[B28] Clark HR Goyder E Bissell P Blank L Walters SJ Peters J A pilot survey of socioeconomic differences in child-feeding behaviours among parents of primary-school children Public Health Nutr 2007 11 10 1030 1036 18093351 10.1017/S1368980007001401

[B29] Hendy HM Williams KE Mother’s feeding practices for children 3–10 years of age and their associations with child demographics Appetite 2012 58 2 710 716 10.1016/j.appet.2012.01.011 22269792

[B30] Hardy LL King L Hector D Lloyd B Weight status and weight-related behaviors of children commencing school Prev Med 2012 55 5 433 437 10.1016/j.ypmed.2012.09.009 22995371

[B31] Dekovic M Rispens J Vaders en de opvoeding en ontwikkeling van kinderen Een inleiding. (Fathers and child rearing and evelopment – an introduction) Kind en adolescent 1998 19 1 39 40 10.1007/BF03060680

[B32] Central Committee on Research Inv. Human Subjects [ http://www.ccmo.nl/nl/uwonderzoek-wmo-plichtig-of-niet]

[B33] BMI classification [ http://apps.who.int/bmi/index.jsp?introPage=intro_3.html]

[B34] Keij I Standaarddefinitie allochtonen. (Standard definition of ethnic minorities) Index 2000 10 24 25

[B35] Sociaal Cultureel Planbureau (SCP) (The Netherlands Institute for Social Research) Statusscores (Status scores) http://www.scp.nl/content.jsp?objectid=default:20133

[B36] Verweij A Scholing en opleiding: indeling opleidingsniveau. (Education and training: categorization of educational levels) 2008 Bilthoven: National Institute for Public Health and the Environment

[B37] Brink van den CL Blokstra A Hoeveel mensen hebben overgewicht of ondergewicht? (How many people are overweight or underweight?) 2013 [ http://www.nationaalkompas.nl/gezondheidsdeterminanten/persoonsgebonden/overgewicht/hoeveel-mensen-hebbenovergewicht/]

[B38] Campbell KJ Crawford DA Hesketh KD Australian parents’ view on their 5–6-year old children’s food choices Health Promot Int 2006 22 1 11 18 17043065 10.1093/heapro/dal035

[B39] Birch LL Development of food preferences Annu Rev Nutr 1999 19 41 62 10.1146/annurev.nutr.19.1.41 10448516

